# Is It Worthwhile to Fully Evaluate the Stomach in Every Ultrasound Examination of the Abdominal Cavity?

**Published:** 2011-03-30

**Authors:** M. Goudarzi, J. Navabi, Gh. Salimi

**Affiliations:** 1 Assistant Professor, Department of Radiology, Kermanshah University of Medical Sciences, Kermanshah, Iran; 2 Assistant Professor, Department of Internal Medicine, Kermanshah University of Medical Sciences, Kermanshah, Iran

**Keywords:** Ultrasonography, Abdominal Cavity, Gastrointestinal Endoscopy, Sensitivity, Specificity

## Abstract

**Background/Objective:**

To evaluate the usefulness of abdominal sonography in the fasting state with no hypotonic agents in the detection and exclusion of gastric lesions.

**Patients and Methods:**

One-hundred patients with normal upper gastrointestinal endoscopy, 94 patients with a major gastric abnormality (including 59 intraluminal tumors, three submucosal masses, 29 ulcers, two polyps and one hypertrophied gastric mucosa) and 75 patients with minor gastric abnormalities (mainly gastritis) were enrolled into the study.

**Results:**

Of the 100 normal patients, ultrasound showed four false positive results with 96% specificity of the examination. Within the major gastric lesion group, ultrasound was true positive in 55 of 59 tumors, 15 of 29 ulcers, three of three submucosal masses and the case of giant gastric mucosa. It was negative in the detection of gastric polyps. It could detect only 8% of minor gastric abnormalities.

**Conclusion:**

Abdominal sonography in the fasting state, if carefully performed, is sufficiently accurate in detection and exclusion of major gastric lesions. Therefore, although it cannot replace endoscopic and barium studies of the stomach, careful evaluation of the stomach is recommended in every sonographic evaluation of the abdominal cavity.

## Introduction

Patients with upper gastrointestinal (GI) abnormalities may present with nonspecific symptoms. On the other hand, gastric cancer, in the early stages and when it is surgically curable, usually produces no symptoms and the disease presents usually at advanced stage. Peptic ulcers may also present with a complication without antecedent symptoms.[1][2][3] Although upper GI endoscopy and barium studies are accepted methods for the evaluation of upper GI lesions, it is not unusual to see a gastric lesion during a routine abdominal ultrasound (US) examination. In fact, US is frequently used as a primary diagnostic test for evaluation of patients with nonspecific abdominal complaints and acute abdominal pain.[4][5] To date, many studies have been carried out on the usefulness of US in the diagnosis of gastric lesions, but nearly all of them by means of fluids and/or hypotonic agents to distend the stomach.[6][7][8][9][10][11][12][13][14][15][16][17][18][19][20][21] We performed this prospective study to assess the usefulness of abdominal sonography in the fasting state and without use of hypotonic agents in the evaluation of the stomach, as is usually occurs in a routine abdominal US examination.

## Patients and Methods

During an approximate three-year-period from July 2005 to March 2008, 269 patients with an upper GI endoscopic study in the endoscopic unit of our hospital were referred for US evaluation of the abdominal cavity with focus on the epigastric region. All examinations were performed up to one week after endoscopic studies. All examinations were carried out by the same single radiologist with more than ten years experience in US study of the abdominal cavity who was unaware of the endoscopic findings. Nearly all of the US examinations were done using real time scanners (Aloka SSD 1100-Tokyo-Japan & Medison SonoAce 4800HD-Seoul-Korea) with 3.5 MHz convex transducers. All possible positions, especially the left lateral decubitus position using the left lobe of the liver as a sonic window were applied to evaluate all parts of the stomach as completely as possible. All patients were in the fasting state for at least eight hours. Upper GI barium study was done in some patients before the endoscopic-sonographic evaluation or after that in a few cases. The ethical committee of Kermanshah University of Medical Sciences approved the study. A gastric wall thickness of 5 mm or more was considered as the criterion for pathologic state and the results were compared with the endoscopic findings as gold standard and histopathologic findings in a few cases.

The patients were divided into three groups; namely, group A with no endoscopic abnormality, group B with major endoscopic abnormalities (mainly gastric ulcers and mass lesions) and group C with minor endoscopic abnormalities (mainly gastritis). No case was excluded even in the presence of obesity or gas distention of the bowel loops or the stomach.

## Results

**Fig. 1:**
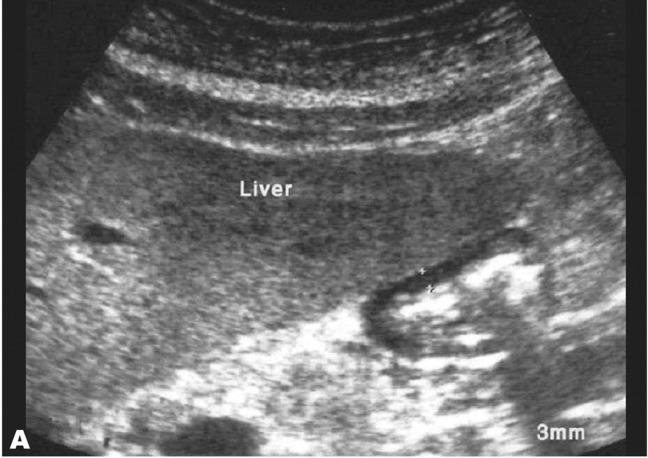
Transverse abdominal ultrasound image in a healthy 38-year-old woman demonstrating normal hypoechoic gastric wall thickness of less than 5 mm.

Within the 100 patients of group A, including 46 men with the mean age of 48 years (18-79) and 54 women with the mean age of 41 years (16-75), US was normal in 96 cases with a gastric wall thickness of less than 5 mm (Fig. 1). Four false positive ultrasound results were seen as 5 mm gastric wall thickness (borderline measurement) with 96% specificity of the study. Two cases of pancreatic mass lesions and a case of acute pancreatitis were found incidentally.

**Fig. 2:**
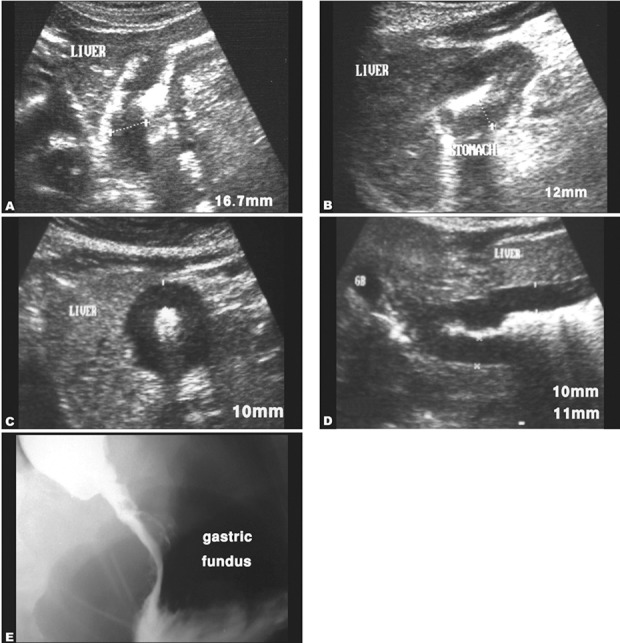
Abdominal ultrasound images showing gastric adenocarcinomas in fundus A, body B and antrum C,D of the stomach. E. The barium meal image of case A

Of the 94 patients of group B, 59 cases had mass lesions, including 36 men with the mean age of 68 years (37-80) and 23 women with the mean age of 64 years (33-84). Forty eight patients had polypoid lesions and 16 cases had ulcerated masses, of which seven patients had gastric outlet obstruction. US was positive in 55 cases, as irregular, usually circumferential gastric wall thickening, ranging from 7 to a maximum of 30 mm (Fig. 2).

**Fig. 3:**
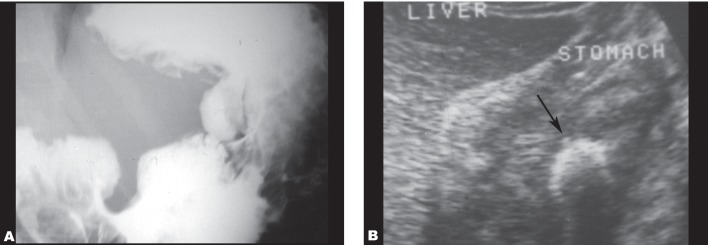
Malignant gastric ulcer in a 55-year-old man.( (A) Shows barium meal in this patient. (B) Abdominal sonography shows thickening of the gastric wall associated with a fixed echogenic area (arrow in B) probably representing the ulcer crater.)

**Fig. 4:**
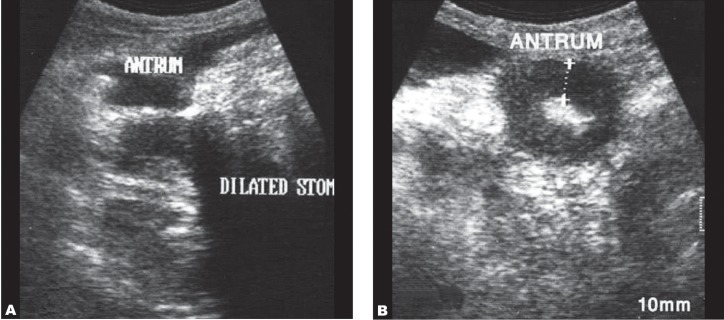
Gastric adenocarcinoma in a 68-year-old man. Longitudinal (A) and transverse (B) abdominal ultrasound images show gastric antrum wall thickening in association with fluid distention in the proximal parts of the stomach.

An echogenic area possibly representing tumor ulceration was seen in three of the ulcerated lesions (Fig. 3). It also demonstrated gastric outlet obstruction in six of the seven patients by demonstrating fluid distention of the stomach (Fig. 4). The size of the missed tumors ranged from a 0.5-1 cm lesion in the gastric antrum to a 2-3 cm lesion in the gastric fundus. US did not show any of the two gastric polyps. US demonstrated all three mural submucosal lesions by showing hypo-isoechoic well defined round-oval mass lesions displacing the echogenic lumen of the stomach. 

**Fig. 5:**
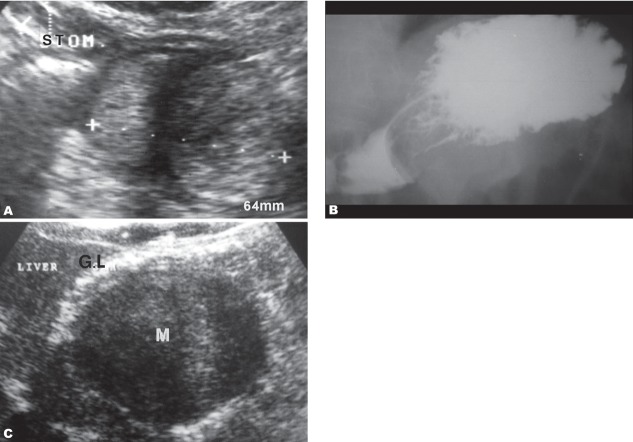
Abdominal ultrasound (A) and barium meal (B) images in a 47year-old lady with gastric wall leiomyoma. In another similar case (C), ultrasound image shows displacement of the gastric lumen (GL) gas by the submucosal mass lesion (M).

**Fig. 6:**
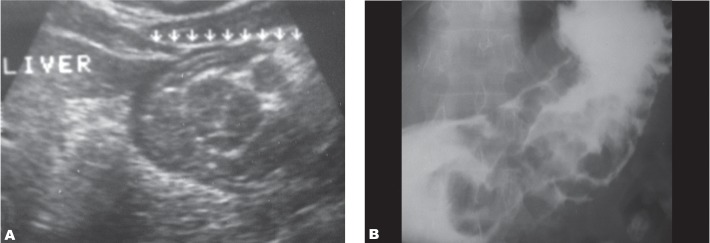
Abdominal ultrasound (A) and barium meal (B) images demonstrating giant hypertrophy of the gastric mucosal folds in a 52-year-old man, probably related to chronic alcohol consumption.

**Fig. 7:**
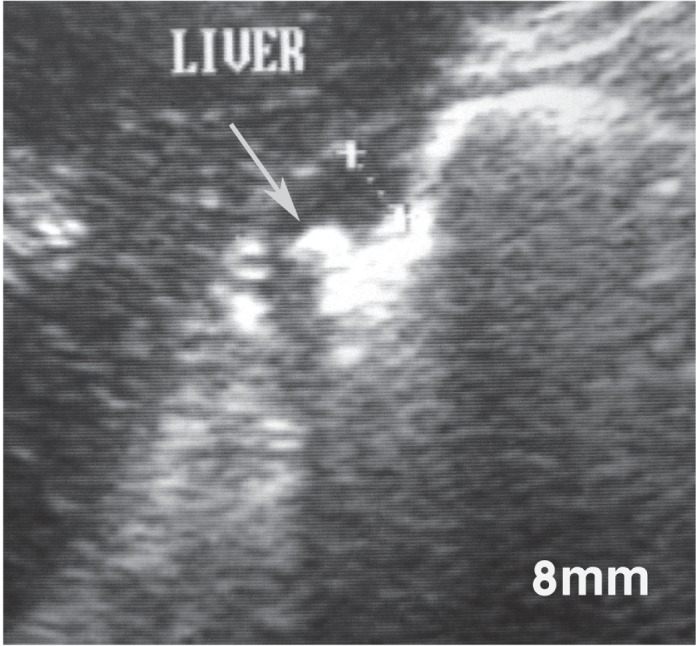
Abdominal ultrasound image in a 48-year-old female with benign lesser curvature gastric ulcer showing thickening of the gastric wall and a niche-like echogenicity (arrow), probably representing the ulcer carter.

Barium study was done in all of them after endoscopy and US study (Fig. 5). In the single case of giant gastric mucosal hypertrophy, US showed hypoechoic thick mucosal folds with linear echogenic mucosal interface in between (Fig. 6). Of the 29 patients with gastric ulcers, US was positive in 15 cases, nine men with the mean age of 58 years (10-75) and six women with the mean age of 55 years (47-70). In all the positive results, there was a localized gastric wall thickening of 6 to 12 mm, in three cases associated with a niche like echogenecity extending from the inner echogenic gastric lumen into the thickened gastric wall (Fig. 7). Two ulcers were malignant, one presenting as multiple ulcerations and the other as a 1-2 cm lesion. Of the 14 false negative cases, including 11 men with the mean age of 62 years (45-85) and three women with the mean age of 56 years (42-75), one was a linear ulcer and two presented with acute GI bleeding.

Within group C patients, including 38 men with the mean age of 46 years (19-76) and 37 women with the mean age of 40 years (15-77), US was positive in only 8% of the cases with gastritis, all of them as borderline measurements (5 mm). Among the three cas es of erosive gastritis with GI bleeding, US was negative. In a case of hiatal hernia, a fixed pouch of gastric fundus with air within was noted in a gastroesophageal junction area.

## Discussion

As other parts of the GI tract, US appearance of the normal stomach is an irregular echogenic center surrounded by a hypo-echoic rim, representing its lumen and wall, respectively.[6] The thickness of this rim has been reported to be 5.107±1.1 mm, and 7 mm as the highest value in the normal subjects.[7] The typical US appearance of a gastric wall abnormality is a thick hypoechoic area surrounding an echogenic center (pseudokidney or target lesion).[6] Although some authors have reported specific US patterns in different gastric abnormalities[8][9][10] and even a scoring system or a measurement threshold has been suggested for differentiation of benign and malignant gastric abnormalities by US,[11][12] gastric wall thickening is usually a nonspecific finding and might be the result of neoplastic or non-neoplastic conditions.[10] In this study, most gastric neoplasms appeared as circumferential and all gastric ulcers as localized gastric wall thickening, but it should be noted that in either case, endoscopy or barium studies are required to confirm the diagnosis. Therefore, as Lorentzen et al. stated,[6] the primary benefit of abdominal US is in the detection of the abnormality and providing information about its size, location, shape and relation to other organs if possible. By excluding the results of group C (insignificant gastric abnormalities), the specificity of 96% and overall sensitivity of 78.7% for major gastric lesions show that this procedure, if carefully performed, permits detection of gastric lesions in a high percentage of patients in the fasting state without the use of fluids or hypotonic agents. Our results are in agreement with those of others who evaluated the stomach with fluids and/or hypotonic agents.[10][13][14] Worlicek et al., in a series of 68 patients with a gastric wall abnormality and 39 normal subjects with the use of 500-1000 cc orange juice and a hypotonic agent, reported an overall sensitivity of 82.4% and specificity of 94.9%.[10] It should be noted that they studied some parts of the stomach with a 5MHz transducer with a higher resolution in comparison with the 3.5 transducers in our study. In a retrospective study of 59 cases of abnormal gastric wall thickening on abdominal US by the use of 3 and 5 MHz transducers with and without the use of fluids, Lorentzen et al. showed that 46 cases were pathologic and eight cases were false positive.[6] Because of difference in methodology, complete comparison of these two studies is not possible, however, as in our study, they stated that gastric wall thickening on abdominal US indicates an abnormality in a high percentage of the patients (86% in their report). Although Morinez et al. could detect 19 out of 20 gastric tumors by the fluid-filled stomach technique and a 5 MHz probe,[15] the sensitivity of 93% in showing gastric neoplasms in our study was higher than other studies that studied the stomach with or without the use of fluids.[10][12][14] One explanation for this higher sensitivity might be the higher stage of the tumors we studied, our higher experience based on previous studies, more careful examination or a combination of all the mentioned factors. Our false negative cases were lesions as small as 1-2 cm in contrast to false negative tumors as large as 8 cm in some reports.[14] It should be noted that although there are such false negative large tumors, cases of early gastric cancers have also been detected in some studies.[10][12] We showed two malignant gastric cancers presenting as small ulcerations, but one limitation of our study was the lack of follow-up of gastric cancers for determination of the stages of the disease. However, these findings suggest a potential role for abdominal sonography in the screening of gastric cancers.

In contrast to our study, gastric polyps have been detected in studies with the use of fluids to distend the stomach.[10][16] We think it is because gastric polyps are usually benign lesions [17] without wall infiltration or edema, so they cannot be detected in the absence of fluid distention of the stomach.

Similar to our study, gastric submucosal-mural lesions have been detected in nearly all series by filling the stomach with fluids.[8][10][18]A sonographic sign has also been described as continuity of gastric layers on the mucosal surface of the lesions.[8] We could not detect such findings in our cases but instead we showed displacement of echogenic gastric lumen in all the cases.

As gastric mass lesions, reasonable sensitivities have been reported on the US evaluation of gastric peptic ulcer disease with the fluid-filled stomach technique.[9][10][13]A dish-shaped niche has been reported by some authors in gastric ulcers.[9][10] We could not detect this finding because of the absence of fluid within the stomach, but we showed a niche like echogenicity extending from the inner echogenic gastric lumen into the thickened gastric wall in about one fourth of our patients. In all positive cases of gastritis, the gastric wall thickness was borderline, in contrast to Joharjy et al.’s study[13] that detected mild gastric wall abnormality (6-8.5mm) in three out of six patients with gastritis and/or duodenitis and severe wall abnormality (more than 8.5 mm) in one of them. However, they did not mention how many of them were cases of pure gastritis. A sensitivity of 55% in the detection of gastric ulcers in our study shows that abdominal sonography in the fasting state is not sufficiently accurate in the detection of these lesions, but it is worth to note that we performed most US examinations immediately after endoscopy, especially in cases with acute GI bleeding and because in the upper gastrointestinal endoscopy, air is insufflated to distend the stomach, it might have interfered with our results, especially in small lesions such as gastric ulcers.

In conclusion, sonography of the abdominal cavity in the fasting state is a sensitive and specific modality in detecting or excluding gastric abnormalities. It is a rapid, available and noninvasive modality that is frequently used as the first diagnostic tool in the evaluation of patients with abdominal complaints, so the radiologist or sonographer should be familiar with its ability to detect gastric lesions at an earlier stage. On the other hand, sonography may be used as a supplementary diagnostic procedure to upper GI barium studies in equivocal cases, follow up of disease activity in patients with known gastric disease [13] and finally reserved for the evaluation of patients who are uncooperative for endoscopy or upper GI barium studies.
